# Advances in immunotherapy for thyroid malignancies: from molecular targets to clinical outcomes

**DOI:** 10.3389/fmed.2026.1754058

**Published:** 2026-02-12

**Authors:** Shuo Lv, Jinbao Wang, Guohao Chen, Yongshun Wang, Naiqing Liu

**Affiliations:** 1 Shandong Second Medical University, Weifang, China; 2 Linyi Central Hospital, Linyi, China; 3 Linyi People’s Hospital, Linyi, China

**Keywords:** anaplastic thyroid carcinoma, BRAFV600E mutation, CAR-T lymphocytes, CTLA-4 pathways, immune checkpoint inhibitors, LAG-3, medullary thyroid carcinoma, neoantigens

## Abstract

**Background:**

Thyroid cancers comprise a diverse collection of endocrine tumors, notably papillary, follicular, medullary, and anaplastic carcinomas, each differentiated by their molecular alterations, clinical behavior, and responsiveness to therapies. Current treatment algorithms of surgical resection, radioiodine treatment, and selective small-molecule inhibitors, although effective for many cases, confront significant limitations, particularly in anaplastic and advanced medullary tumors, where resistance to conventional agents correlates with diminished prognosis, thereby demanding the exploration of innovative therapeutic strategies.

**Purpose:**

This article reviews contemporary immunotherapy-directed interventions for thyroid cancers, highlighting the elucidation of actionable tumor antigens, the reengineering of the immunologic tumor microenvironment, and the ongoing efforts to translate these laboratory findings into practicable, evidence-based clinical protocols.

**Key findings:**

Recent studies underscore the critical efficacy of immune checkpoint inhibitors targeting the PD-1/PD-L1 and CTLA-4 pathways in select populations of anaplastic thyroid carcinoma (ATC), medullary thyroid carcinoma (MTC), and PD-L1-expressing differentiated thyroid cancers. Next-generation immune modulators, specifically inhibitors directed against LAG-3 and TIM-3, are being evaluated in combinatorial frameworks. Vaccines engineered to elicit responses against the BRAF^*V*600*E*^ mutation, RET/PTC fusions, and additional neoantigens have shown promising immunogenic profiles in preliminary trial cohorts, while adoptive transfer methodologies, including tumor-infiltrating lymphocyte (TIL) mobilization and engineered CAR-T lymphocytes, are progressing through preclinical and early-phase clinical benchmarks. Concurrently, oncolytic viral vectors are being harnessed to amplify neoantigen liberation and, consequently, to amplify systemic immunity. When immunotherapeutic modalities are judiciously aligned with tyrosine kinase inhibitors (TKIs) or radiotherapeutic regimens, cumulative anti-tumor effects are accentuated, purportedly through mechanisms such as immunogenic cell death induction and the reprograming of immune-tolerant tumor ecosystems.

**Conclusion and future perspective:**

Immunotherapy is set to transform the treatment paradigm for thyroid cancers, although remaining hurdles, the disquietingly low baseline immunogenicity of differentiated tumors, the rapid, capricious emergence of resistance, and complex immune-related endocrine toxicities, must be systematically addressed. Success in this arena will hinge on utilitarian biomarker-based cohort selection, the discovery of fresh immunogenic epitopes, and the meticulous design of synergistic treatment combinations. The synergistic leverage of genomic, transcriptomic, and immune landscape dissection, coupled with cutting-edge engineered lymphocyte platforms and engineered oncolytic vectors, may finally position immunotherapy as an unassailable pillar of bespoke medicine for advanced thyroid carcinomas.

## 1 Introduction

The risks of thyroid cancers are reported in smokers, alcoholics, obese and people with low physical activity and are found to be higher in women than men (1), and thyroid malignancies rank as the most frequently diagnosed endocrine cancers (2, 3), representing an assemblage of histological subtypes that includes papillary thyroid carcinoma (PTC), follicular thyroid carcinoma (FTC), medullary thyroid carcinoma (MTC), and anaplastic thyroid carcinoma (ATC) (4–8). Differentiated carcinomas, notably PTC and FTC, typically enjoy an auspicious prognosis under existing management regimens, yet clinically aggressive phenotypes, primally ATC and high-stage MTC, continue to confer dismal survival figures even in the context of multimodal treatment (9, 10). Longitudinal epidemiological data signal an ascending global thyroid cancer incidence, a trend ascribed in part to enhancements in diagnostic accuracy (11); conversely, mortality figures for advanced disease have scarcely budged, thereby compelling the search for emergent, efficacious therapeutic alternatives (12, 13). A summary of the origin, incidence, and prevalence of several classes of thyroid cancers is given in Table 1 (14). However, a distinct dichotomy exists: while rare subtypes like ATC often exhibit an inflamed phenotype responsive to checkpoint blockade, the more common differentiated thyroid cancers (DTCs) are typically immunologically ‘cold’ and resistant to monotherapy.

**TABLE 1:** Classification and prevalence of various types of thyroid cancers (14).

Tyroid tumors		Thyroid origin	Incidence (%)	Mutations	5-year survival
Differentiated TC	PTC	FC Follicular cells	80	RET rearrangements BRAF, RAS	98%
	FTC		10	RAS	95%
Poorly differentiated TC			2-15	BRAF, EIF1AX, RAS, TERT RET rearrangements	66%
ATC			1	BRAF, TP53, RAS, TERT RET rearrangements	12%
MTC		C cells	1-3	RET mutations	65%

The present clinical toolkit for thyroid malignancies consists of complete surgical excision, radioactive iodine (RAI) (^131^I) detoxification (15–17), suppression of thyroid-stimulating hormone (TSH) (18), external-beam irradiation (19), and, for specific situations, targeted molecular compounds such as tyrosine kinase inhibitors (TKIs) (20, 21) that inhibit RET, BRAF, or VEGFR signaling (5, 22–26). Such modalities have augmented survival and reduced recurrence in carefully selected populations (12). Yet, their overall benefit is limited by either inherent or emergent resistance, inadequate control of RAI-refractory disease, and the accumulation of toxic sequelae that diminish life quality (27). The severity of these shortcomings is accentuated in ATC, for which median survival rarely surpasses 12 months, and in advanced MTC, for which prolonged disease stabilization after TKI therapy is seldom observed (22, 28, 29).

Immunotherapeutic strategies have gained traction as potentially beneficial interventions across a spectrum of neoplasias, operating by potentiating the endogenous immune repertoire to detect and eliminate malignant cells (30–32). The justification for deploying such therapies in thyroid oncology is supported by evidence that the thyroid tumor microenvironment (TME) contains measurable immune cell populations, expresses inhibitory checkpoint ligands (33, 34), and harbors genetic alterations capable of yielding neoantigens that could provoke an immune attack (35). Notwithstanding, thyroid neoplasms demonstrate heterogeneous immunogenic landscapes (30), with ATC manifesting an elevated tumor mutational burden (TMB) (6, 36) and a more pronounced inflammatory infiltrate when contrasted to the relative immunological quiescence that typifies differentiated thyroid cancers (DTCs) (37–39).

Recent advancements in immuno-oncology, particularly the refinement of immune checkpoint inhibitors (ICIs), adoptive cell transfer platforms, therapeutic vaccines, and oncolytic viral agents, have inaugurated promising strategies for the management of advanced, treatment-refractory thyroid neoplasms (40–43). Clinical investigations have reported notable efficacy of programed death-1/programed death-ligand-1 (PD-1/PD-L1) and cytotoxic T-lymphocyte-associated protein-4 (CTLA-4) antagonism in ATC and niche cohorts of MTC, while combination regimens incorporating checkpoint blockade (44–48), TKIs radiotherapy have elicited complementary effects via TME modification and the elicitation of immunogenic cell death (20, 21, 49, 50). Concurrent preclinical investigations have pinpointed further immune modulators, including LAG-3 and TIM-3, as strategic nodes for dismantling adaptive immune resistance (42, 44, 51).

This review affords an integrative overview of immunotherapy evolution within thyroid oncology, traversing the molecular and cellular lexicon of immune evasion, the dissection of attainable immunologic targets, and the iterative translation of these insights into therapeutic protocols (52–54). We delineate the unique immune microenvironments characterizing thyroid cancer subtypes, assess the therapeutic viability of novel immunomodulatory agents, and explore the prospective incorporation of immunotherapy into multimodal treatment regimens (31, 49, 55–57). We also scrutinize prevailing obstacles, encompassing immune-related endocrinopathies, the need for robust biomarkers delineating patient eligibility, and the evolving mechanisms underpinning therapeutic (58–60). Finally, we delineate strategic research trajectories aimed at amplifying the clinical benefit of immunotherapeutic interventions across this heterogeneous cadre of endocrine neoplasms (53, 61, 62).

## 2 Tumor immunology of thyroid malignancies

### 2.1 Thyroid tumor microenvironment

**FIGURE 1:**
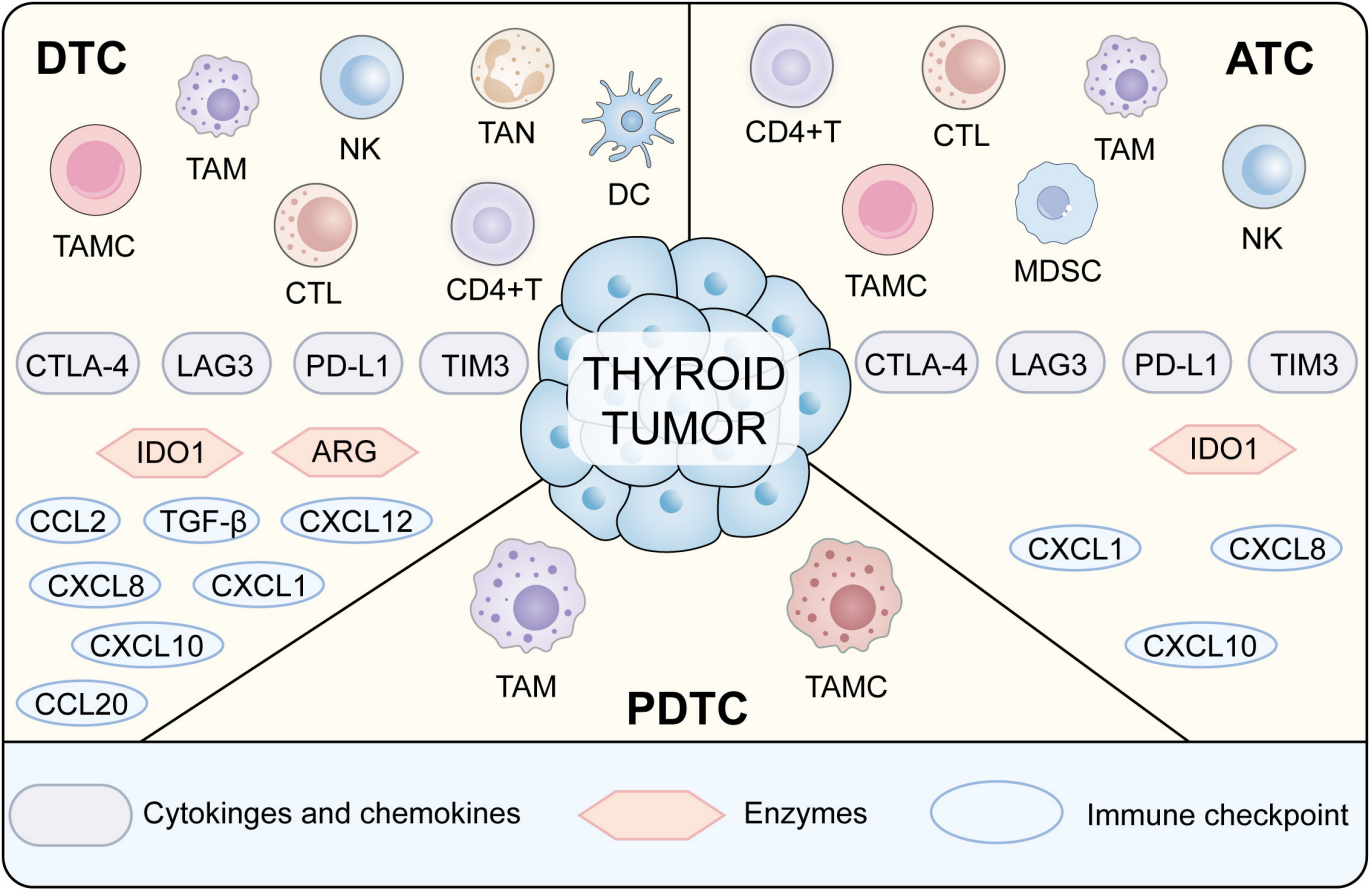
A schematic representation of thyroid tumor microenvironments (69). Details are given in the text.

The microenvironment surrounding thyroid tumors is a highly organized and ever-evolving milieu that integrates neoplastic cells, immune constituents, stromal components, blood vessels, and an array of cytokines (31, 63). The composition and functionality of these immune constituents differ according to the specific tumor histotype and the broader disease trajectory. Tumor-infiltrating lymphocytes (TILs), particularly populations of CD8^+^ cytotoxic and CD4^+^ helper T cells, serve as principal agents of immunological assault against the neoplasm (47, 64); yet, their effective firepower is commonly undermined by a spectrum of immunosuppressive cells, notably regulatory T cells (Tregs), myeloid-derived suppressor cell (MDSC) accumulations, and tumor-associated macrophages (TAMs) (33, 36, 65–67). The more malignant forms, particularly ATC, manifest a striking enrichment of M2-polarized TAMs that, through the secretion of immunomodulatory cytokines and inductive signals for neovascularization, effectively abbreviate tumor immune surveillance (6, 68). Natural killer (NK) cell populations are detectable, yet their cytotoxic functionality frequently diminishes, a setback attributable to the tumor’s ability to enact selective immune evasion (65). As depicted in Figure 1, the transition from DTC to ATC correlates with a shift from a pauci-immune environment to one characterized by heavy macrophage infiltration and complex cytokine networks.

The cytokine and chemokine landscape within the thyroid TMEs critically orchestrates the recruitment and functional modulation of immune cells (70, 71). Increased secretion of immunosuppressive cytokines such as interleukin-10 (IL-10) and transforming growth factor-beta (TGF-β) drives T-cell energy, whereas chemokine interactions along the CXCL12/CXCR4 axis promote tumor cell dispersal and metastatic dissemination (70, 72, 73). Concurrently, inflammatory mediators like IL-6 and TNF-α may paradoxically sustain tumor progression via prolonged inflammatory stimulation (74). Together, these factors craft an immune-privileged microenvironment that supports immune energy and tumor endurance (39).

### 2.2 Immune evasion mechanisms

Thyroid neoplasms adopt multiple immune evasion tactics to escape surveillance and destruction (75). A signature strategy involves selective downregulation of major histocompatibility complex class I (MHC-I) glycoproteins, attenuating the capacity of tumor cells to present neo-antigens to cytolytic T lymphocytes (34). This phenomenon is especially marked in poorly differentiated and anaplastic variants, leading to diminished detection by the adaptive immune compartment (70).

A further important mechanism comprises the increased expression of immune checkpoint ligands, particularly PD-L1, on both malignant thyroid cells and the immune infiltrate (76). The interaction of PD-L1 with PD-1 receptors on effector T cells dampens cytotoxic activity and contributes to T-cell functional exhaustion (70). In ATC, elevated PD-L1 levels are often detected and are associated with a TME that is characterized by an inflammatory signature, both marking a potential target for checkpoint inhibition and reflecting adaptive resistance of the immune compartment (6, 33).

Simultaneously, the macrophage switch toward the M2 phenotype generates a milieu that favors tumor tolerance through the secretion of IL-10, vascular endothelial growth factor (VEGF), and various matrix metalloproteinases (MMPs), all of which promote tumor expansion and secondary spread (71). These pathways further undermine T-cell expansion by degrading tryptophan and generating metabolites that inhibit effector functions (34). Collectively, these signals create a permissive immune environment that reduces the likelihood of immune-mediated tumor rejection and hinders the progression of spontaneous regression (55).

#### 2.3 Immunogenic variations among thyroid cancer subtypes

**FIGURE 2:**
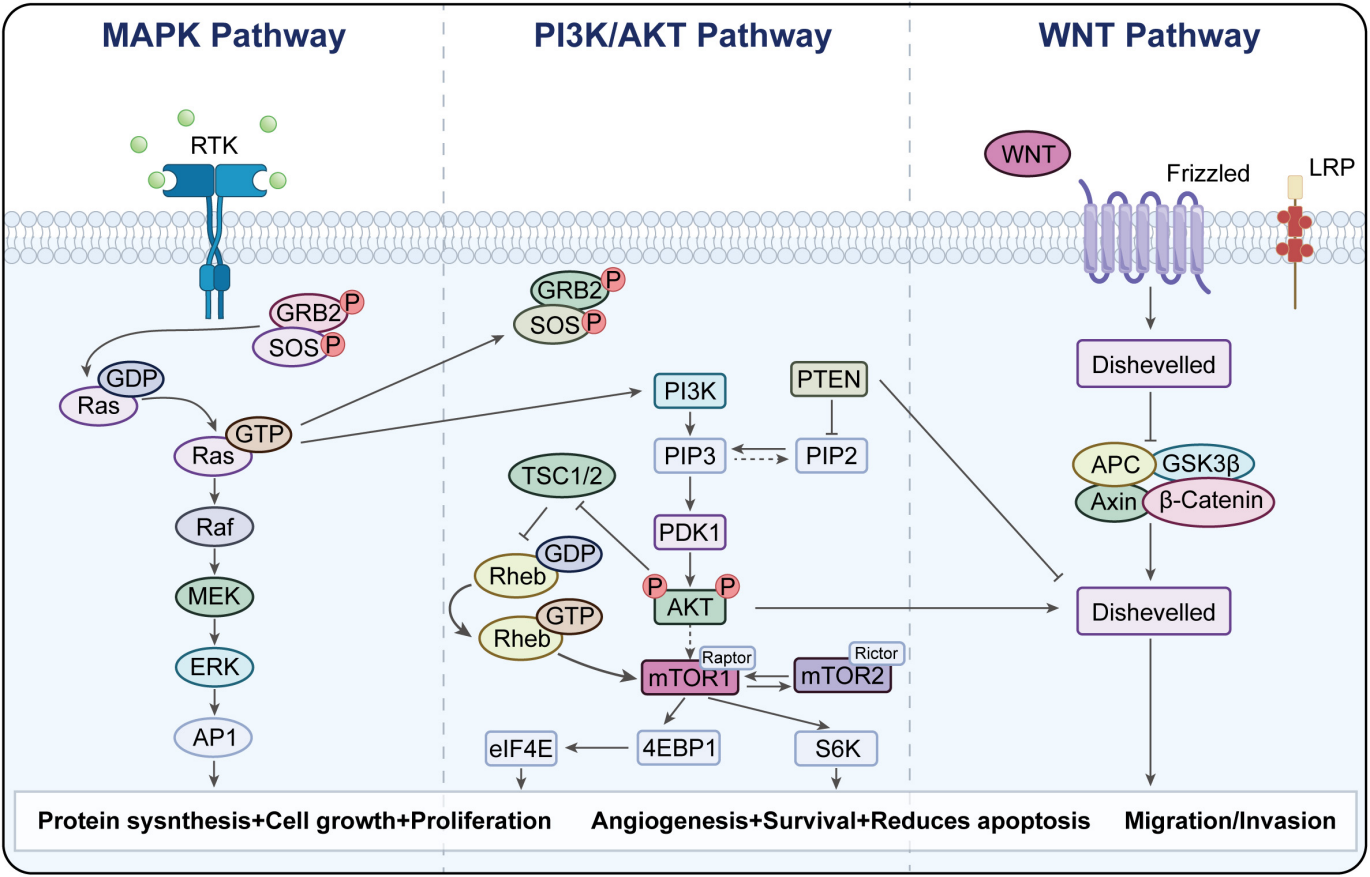
Genetic modifications affecting MAPK, PI3K and WNT signaling paths in thyroid cancer (39).

The immunogenic landscape of thyroid neoplasms is markedly diverse across subtypes (39). Various self-explanatory MAPK, PI3K, and WNT signaling pathways affected and modified in thyroid cancers are sketched in Figure 2 (39).

(i)    Papillary thyroid carcinoma (PTC): PTC usually shows moderate immunogenicity, evidenced by the presence of TILs and some cases of PD-L1 expression, particularly within BRAF^*V*600*E*^ mutant variants (74, 77). The overall mutational frequency is low; however, neoantigen formation can still be significant when specific driver mutations coexist, suggesting potential targets for neoantigen-based vaccination and adoptive cell-transfer therapies (78).
(ii)    Anaplastic thyroid carcinoma (ATC): ATC is distinguished by a substantially elevated TMB, dense immune cell infiltration, and recurrent PD-L1 overexpression (79, 80). The conjunction of these immunological characteristics and the synthesis of pro-inflammatory cytokines identifies ATC as an attractive tumor for immune checkpoint blockade, especially when paired with selective kinase inhibitors to amplify the immunologic response (6, 81).
(iii)    Medullary thyroid carcinoma (MTC): Originating from the parafollicular C cell lineage, MTC exhibits a neuroendocrine phenotype with moderate levels of immune cell presence. PD-L1 expression is less common compared with ATC, yet activated RET mutations and the immunogenicity of calcitonin create specific immunologic targets (25, 82). The particular biology of MTC warrants customized immune-intervention strategies, potentially employing tumor-specific peptide vaccines or engineered T-cell receptor therapies (50).
(iv)    Follicular thyroid carcinoma (FTC): FTC is the second most common thyroid carcinoma, metastasizes distantly with poorer outcomes, is subcategorized as minimally invasive, encapsulated, angioinvasive and widely invasive, and its prognostic factors have been determined in various studies (7, 83). Studies have shown that FTC invasive tumor cell clusters markedly overexpress genes linked with pathways interacting with the extracellular matrix (ECM) remodeling and epithelial-to-mesenchymal transition (EMT) (84). The role of immunotherapy in the management of FTC has been extensively reviewed (85).

Awareness of these immunologic profiles, stratified by thyroid carcinoma subtype, is critical for developing the next generation of personalized immunotherapy (29). Tailored approaches can enhance therapeutic effectiveness while reducing the risk of immune-related adverse events (31).

## 3 Molecular targets for immunotherapy

### 3.1 Immune checkpoints

**FIGURE 3:**
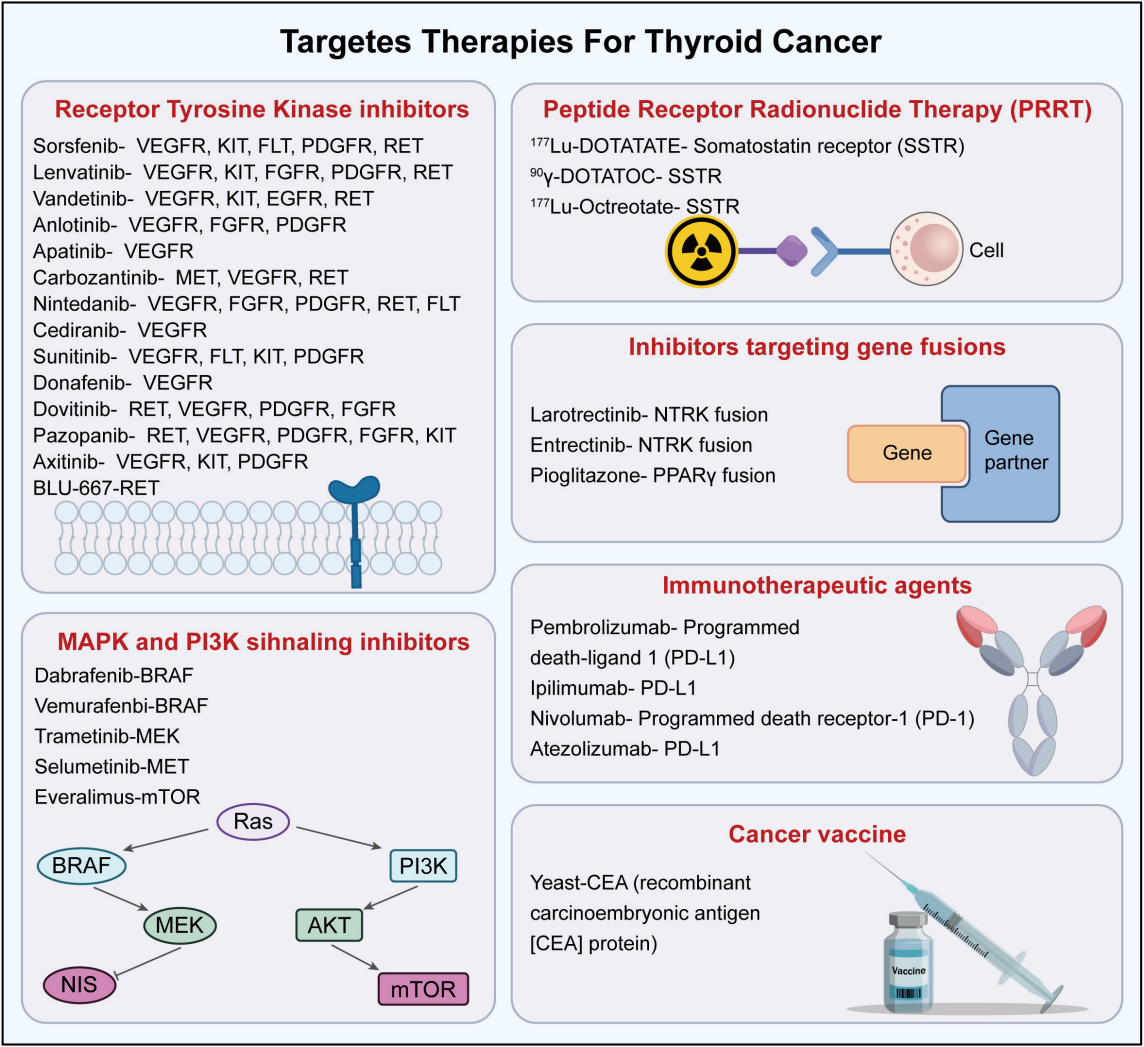
A summary of various targeted therapies for the treatment of thyroid cancers (29).

Immune checkpoint molecules represent critical inhibitory pathways that preserve self-tolerance while fine-tuning the intensity of immune reactivity, thereby shielding normal tissues from collateral damage (55). In thyroid malignancies, neoplastic cells exploit these pathways to evade detection and destruction by the immune system (29). A summary of the molecular targets and targeted therapies is described in Figure 3 which summarized the landscape of targeted therapies, highlighting the distinction between established receptor tyrosine kinase inhibitors and the expanding repertoire of immunotherapeutic agents.

(i)    Programed Death Protein-1 (PD-1) and its Ligand: PD-1 is found on activated T cells, B cells, and NK cells, and its ligand, PD-L1, is frequently upregulated on both tumor cells and immune cells within the tumor microenvironment (70, 86). In ATC, rising PD-L1 levels have been linked to more aggressive disease, increased mutation burden, and a peritumoral inflamed microenvironment (55). Antibody-mediated blockade using agents such as pembrolizumab or nivolumab has been shown to reinvigorate T-cell cytotoxicity and produce notable efficacy in PD-L1-expressing tumors (76).
(ii)    Cytotoxic T-Lymphocyte-Associated Protein 4 (CTLA-4): CTLA-4 is an inhibitory receptor on activated effector T cells and regulatory T cells (Tregs) (42, 65, 74). It inhibits immune activation by competing with CD28 for B7 ligands on antigen-presenting cells. In thyroid cancer, CTLA-4 blockade with ipilimumab is predicted to augment the activation and proliferation of tumor-reactive T-cell clones, yet its efficacy is still being ascertained, most frequently in stratified combination regimens with PD-1/PD-L1 inhibitors to leverage complementary mechanisms of tumor immune evasion (44, 45, 87).
(iii)    Emerging Negative Regulators (LAG-3, TIM-3, TIGIT): LAG-3 downregulates T-cell blastogenesis and cytokine production following antigen encounter, whereas TIM-3 expression marks T cells subjected to chronic antigenic stimulation, curtailing their effector functions (42, 51). TIGIT inhibits NK and T-cell cytotoxicity by displacing CD226 from the poliovirus receptor (PVR/CD155) (88). All three pathways are now viewed as adaptive immune evasion mechanisms that upregulate following PD-1/PD-L1 inhibition (50). Their identification as redundantly activated circuits in thyroid cancer underscores their utility as rational co-targets in immunotherapeutic strategies aimed at overcoming treatment-resistant disease (44).

To clarify the translational status of these molecular targets, they can be categorized into three stages of clinical development.

(i)    Clinically Validated Targets: The PD-1/PD-L1 axis currently represents the most mature target, with agents like pembrolizumab showing established efficacy in ATC and PD-L1-positive advanced DTC. Similarly, BRAF V600E and RET alterations are standard-of-care targets for tyrosine kinase inhibitors (TKIs), which are increasingly used in combination with immunotherapy to prime the tumor microenvironment.
(ii)    Investigational Targets: Novel checkpoint targets, including LAG-3, TIM-3, and TIGIT, are under active evaluation in clinical trials, primarily as combinatorial partners to reverse adaptive resistance to PD-1 blockade.
(iii)    Emerging/Preclinical Targets: Tumor-associated antigens such as MUC1 and CEA are currently being explored through CAR-T cell therapies and vaccine platforms. While technically feasible, these approaches are predominantly in early-phase trials or preclinical optimization stages.

### 3.2 Tumor-encoded and tumor-associated antigens

Both classes of antigens provide definitional substrates for immunological recognition and therapy, distinguished by their differential expression on neoplastic versus normal tissues (40). Briefly,

(i)    BRAF^*V*600*E*^ Allele: The BRAF^*V*600*E*^ alteration, a nearly universal event in conventional PTC, yields a distinctive peptide that — can be selectively presented on major histocompatibility complex (MHC) molecules ((35, 77, 89). Clinical efforts now include synthetic peptide vaccines, adoptive T-cell therapy with T-cell receptors specific for the altered sequence, and oncolytic vector systems embedding the mutant neoepitope (40, 41, 50, 90).
(ii)    RET/PTC Fusion Genes: Rearrangements that juxtapose the RET receptor tyrosine kinase with diverse partner loci are hallmark lesions in PTC and inherited MTC (82). Peptides derived from the fusion junction and the resulting abnormal tyrosine kinase (TK) domain are under experimental evaluation as immunogenic targets for prophylactic peptide vaccines and for the engineering of T cells with high-affinity chimeric antigen receptors (40, 91).
(iii)    Neoantigens in high TMB ATC: ATC often exhibits a markedly elevated TMB, resulting in a spectrum of novel, immunogenic epitopes that can prime T-cell activity, especially in concert with immune checkpoint inhibitors (6, 36, 92).

### 3.3 Oncofetal and neoantigenic targets

Oncofetal antigens, normally confined to fetal development and re-expressed in neoplasia, afford avenues for immunotherapy with narrow off-target toxicity, as summarized.

(i)    Mucin 1 (MUC1): This heavily O-glycosylated transmembrane glycoprotein is aberrantly upregulated in multiple thyroid malignancies and is amenable to targeted intervention through monoclonal antibodies, therapeutic vaccines, and CAR-T cell strategies (40, 76).
(ii)    Carcinoembryonic Antigen (CEA): Originally characterized in colorectal tumors, CEA is also detectable in subsets of thyroid neoplasms, particularly MTC, and can be harnessed for CEA-specific T-cell engineered approaches (93).
(iii)    Glycoprotein 2 (GP2): This transmembrane glycoprotein 2 is implicated in MTC pathobiology, and data are accumulating to position it as an attainable target for peptide-based or gene-based vaccination protocols (76).

#### 3.4 Cytokine and chemokine pathways

Cytokine and chemokine networks sculpt the immune microenvironment in thyroid cancers, modulating tumorinerizing processes and dictating the efficacy of therapeutic interventions (70, 94) as mentioned below.

(i)    IL-10 and TGF-β: Both cytokines create an immunosuppressive milieu by impairing antigen presentation, attenuating cytotoxic T-cell activity, and fostering regulatory T-cell accumulation, thereby marking them as rational candidates for therapeutic blockade (70).
(ii)    The CXCL12/CXCR4 signaling axis is integral to the translational and metastatic phases of malignancy, governing tumor cell mobilization and the selective recruitment of immunosuppressive myeloid cells to the TME (72, 95). Inhibition of CXCR4 through pharmacological antagonists may augment the therapeutic impact of ICIs by perturbing these supportive signaling networks (76).
(iii)    Pro-inflammatory cytokines, including IL-6 and TNF-α, long regarded as facilitators of tumor progression, can be pharmacologically tuned to drive the TME toward a more favorable immune milieu (65). When such modulation is strategically combined with immune checkpoint blockade, the possibility of reversing protumor polarizations and unleashing cytotoxic T-cell responses is enhanced (70, 96).

## 4 Immunotherapeutic strategies in thyroid cancer

### 4.1 Immune checkpoint inhibitors

**FIGURE 4:**
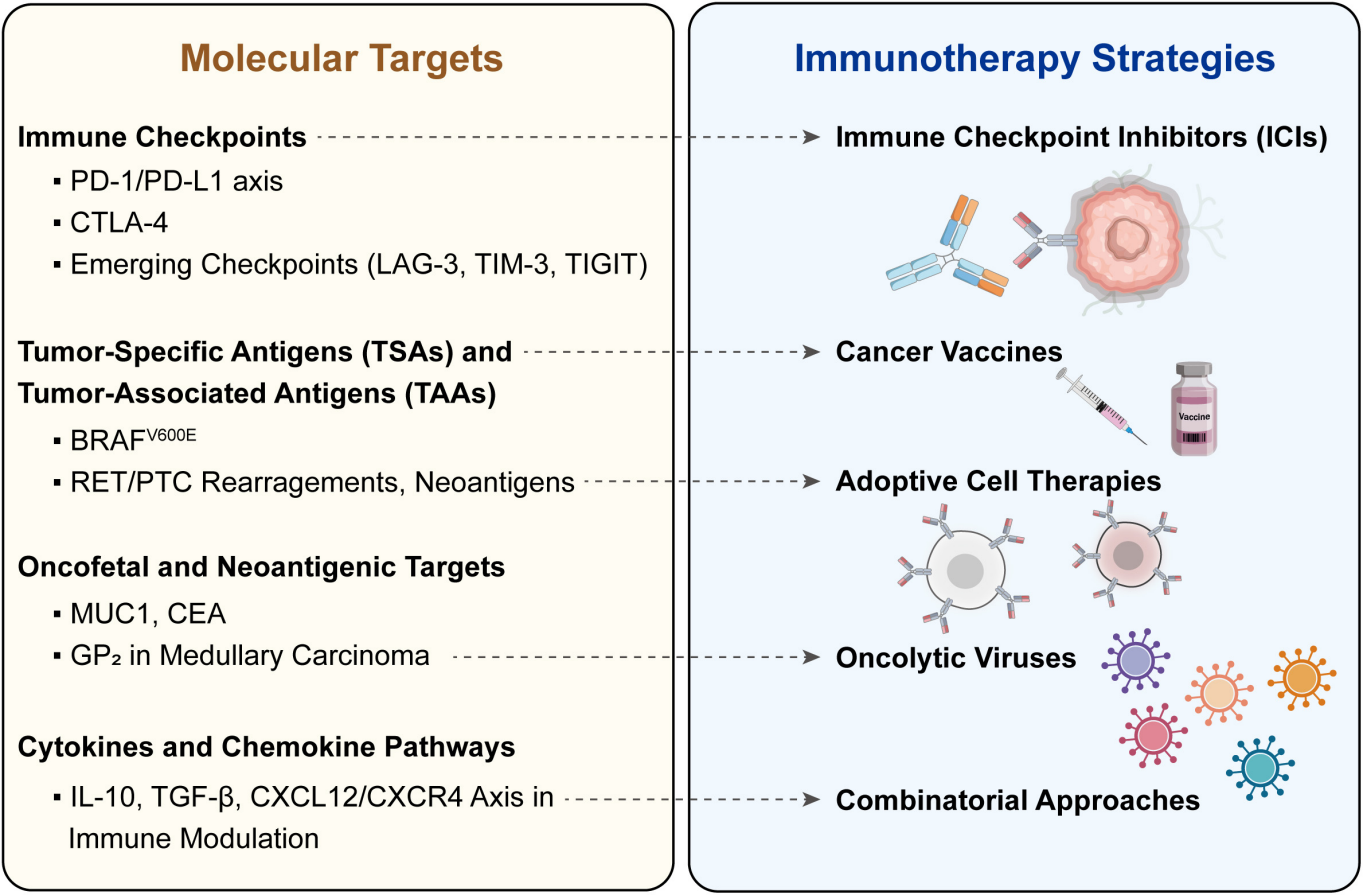
Molecular targets and immunotherapy strategies in thyroid cancers.

Immune checkpoint blockade has established itself as a cornerstone of modern cancer immunotherapy, counteracting suppressive receptor-ligand interactions to restore effective T-cell-mediated anti-tumor immunity (97, 98). In thyroid neoplasms, blockade of PD-1/PD-L1 and CTLA-4 pathways has garnered the most investigational traction (50). A summary of the molecular targets and immunotherapy strategies in thyroid cancers is presented in Figure 4.

(i)    Monotherapy: Pembrolizumab and nivolumab have produced clinically meaningful outcomes in select patients with ATC and MTC characterized by elevated PD-L1 expression (99–101). Among ATC cohorts, those manifesting inflamed TMEs have recorded particularly elevated objective response rates. Cadonilimab therapy has also yielded promising results regarding responses and survival outcomes, with a considerable safety profile (102).
(ii)    Combination with Kinase Inhibitors: Concurrent inhibition of oncogenic signaling pathways and immune checkpoints has been shown to augment the immunogenicity of thyroid tumors (98, 103). The regimen of lenvatinib with pembrolizumab has yielded promising results in ATC (97), a phenomenon attributed to vascular normalization, enhanced antigen presentation, and a decrease in immunosuppressive myeloid cell populations (104, 105).

### 4.2 Cancer vaccines

Therapeutic cancer vaccines are designed to elicit immune responses tailored to tumor-specific antigens (40, 50), such as,

(i)    Peptide-Based Vaccines: Short peptide sequences derived from BRAF^V600E^, RET, and other thyroid tumor-associated antigens have successfully engendered antigen-specific cytotoxic T-cell activation in both preclinical systems and early-phase clinical studies (82, 92, 97).
(ii)    Dendritic Cell Vaccines: Dendritic cells loaded with tumor-associated antigens can activate naĭve T cells, thereby bolstering the immune system’s ability to recognize and attack thyroid cancer cells (106, 107). Preliminary clinical trials in MTC indicate that the approach is technically feasible, although the durability of the immune response and clinical outcomes over the long term have yet to be convincingly demonstrated (91, 108).

### 4.3 Adoptive cell therapies

Adoptive cell therapy (ACT) encompasses strategies that expand, engineer, and reinfuse patient-derived or donor-derived immune cells to achieve robust anti-tumor effects (43, 90). For example,

(i)    Tumor-Infiltrating Lymphocyte Therapy: Lymphocytes extracted directly from tumor tissue can be cultured and reinfused to boost the patient’s existing anti-tumor immunity (64, 109). Despite the technical hurdles posed by tumors with low immunogenicity, such as those with low mutation burdens, this technique could be particularly advantageous in ATC or in PTC cases that show high PD-L1 expression (45, 79).
(ii)    Chimeric Antigen Receptor T Cells: T cells genetically reprogramed with chimeric antigen receptors directed against antigens like MUC1, carcinoembryonic antigen (CEA), or emerging tumor-specific thyroid markers show potential (31). *In vitro* and *in vivo* models have indicated that the effectiveness of CAR-T cells may be markedly improved by concurrent checkpoint blockade, which alleviates suppression from the TME (68).

### 4.4 Oncolytic viruses

Oncolytic viral therapy (OVT) capitalizes on genetically modified, replication-competent viruses that selectively infect and destroy tumor cells (90). The viral replication cycle releases tumor-derived antigens, thereby stimulating systemic antitumor immunity (41). In thyroid cancer models, oncolytic adenoviruses and vaccinia viruses have shown promising effects, particularly against ATC (110). Further gains in antitumor potency can be achieved by engineering the viruses to produce immune-enhancing cytokines such as granulocyte-macrophage colony-stimulating factor (GM-CSF), which can act synergistically with ICIs (50, 70).

### 4.5 Combinatorial approaches

Given the multifactorial underpinnings of immune resistance across thyroid cancers, the development of rationally designed combination strategies is gaining momentum in translational research and clinical practice (58, 108). Briefly,

(i)    ICIs plus targeted therapies: The concurrent blockade of key oncogenic kinases, such as BRAF, RET (82, 92, 97), and VEGFR (111), together with immune checkpoint axes, is postulated to enhance tumor immunogenicity while concurrently dampening the immunosuppressive TME (45, 69).
(ii)    ICIs plus radiotherapy: Ionizing radiation may trigger immunogenic cell death, upregulate MHC expression (89), and promote the recruitment of cytotoxic T lymphocytes to the tumor bed, cumulatively neutralizing tumor-intrinsic immune evasion mechanisms and potentiating checkpoint blockade (47, 49, 95, 112).
(iii)    ICIs plus adoptive cell transfer or therapeutic vaccines: Either sequential or concurrent deployment of ICIs with adoptive T-cell transfer or peptide/protein-based vaccines aims to prolong and sustain T-cell activation while circumventing the adaptive upregulation of immune inhibitory pathways (35, 42, 69, 90).

## 5 Clinical evidence and translational outcomes

### 5.1 Summary of key clinical trials

Multiple Phase I, Phase I/II, and ongoing investigations are systematically evaluating immunotherapy in thyroid malignancies, with a particular focus on advanced disease stages and settings characterized by refractoriness to standard cytotoxic or kinase inhibitor regimens (Table 2) (98, 105, 108, 113).

**TABLE 2:** Summary of immunotherapeutic strategies and clinical evidence stratified by thyroid cancer subtypes.

Subtype	Immunogenic profile and molecular features	Recommended/ investigational strategies	Key clinical evidence and outcomes
Differentiated Thyroid Cancer (DTC)	“Cold” Phenotype: Low tumor mutational burden (TMB) and limited TIL infiltration. Targets: BRAF V600E, VEGFR, PD-L1 (subset).	Combinatorial Approaches: ICI + TKIs (e.g., Lenvatinib) to modulate TME. ICI + Radiotherapy to induce immunogenic cell death.	Modest Monotherapy Efficacy: Pembrolizumab monotherapy showed ORRs of 9–23% in PD-L1 + cases. Combination Promise: Lenvatinib + Pembrolizumab is under evaluation to overcome low immunogenicity.
Anaplastic thyroid cancer (ATC)	“Hot” Phenotype: High TMB, dense immune infiltration, and frequent PD-L1 overexpression. Targets: PD-1/PD-L1, BRAF V600E.	Immune Checkpoint Blockade: Anti-PD-1 monotherapy (Pembrolizumab/Nivolumab). Targeted Combos: ICI + BRAF/MEK inhibitors or Lenvatinib.	Significant Efficacy: ICI + Lenvatinib achieved ORRs approaching 40% with prolonged progression-free survival. Dabrafenib + Trametinib + ICI shows potential in BRAF-mutated ATC.
Medullary thyroid cancer (MTC)	Neuroendocrine Profile: Moderate immune presence; distinctive viral/tumor antigens. Targets: RET mutations, CEA, Calcitonin.	Vaccines and Adoptive Cell Therapy: Peptide/Dendritic Cell vaccines targeting RET/CEA. Synergy with RET inhibitors.	Feasibility Established: Vaccination elicits antigen-specific T-cell responses, though robust tumor regression remains rare. Focus remains on combining immunotherapy with RET-targeted TKIs.

(i)    Immune Checkpoint Inhibitors (ICIs): Pembrolizumab given as monotherapy has revealed preliminary activity in advanced, PD-L1-expression-positive ATC and differentiated thyroid carcinoma, with objective response rates (ORRs) oscillating between 9 and 23% and sustained responses noted in a minority of patients (92, 100). Nivolumab has, in small cohorts, reproduced these efficacy parameters (99, 101). Trials assessing combination strategies, most commonly pembrolizumab partnered with lenvatinib, have reported enhanced ORRs of nearly 40% in ATC, alongside prolonged progression-free survival, as indicated by recently reported early-phase trial data (44, 105, 109). Vibostolimab is an innovative anti-TIGIT antibody that totally restricts CD155 binding and triggers activation of T-cells as well as antigen-presenting cells. Vibostolimab combined with pembrolizumab has been verified in clinical trials (88). Good responses with enhanced survival rates and good safety profiles for cadonilimab therapy have also been demonstrated (102). Dabrafenib and trametinib have proved good therapeutic potential in BRAF^V600E^-mutated ATC (114).
(ii)    Cancer Vaccines: Peptide-based immunotherapeutics engineered to present RET-derived epitopes in MTC achieve robust antigen-specific T-cell priming, but clinically meaningful tumor regression is infrequent, underscoring the need to integrate additional immunomodulatory or cytotoxic modalities to enhance durability of response (40, 43, 82, 90).
(iii)    Adoptive Cell Therapy: While adoption in thyroid carcinoma remains investigational, initial human trials employing CAR T-cells directed against MUC1 or CEA suggest the approach is technically practical and can elicit modest tumor shrinkage, warranting further refinement and combinatorial exploration (43, 115).
(iv)    Oncolytic Viruses: Preclinical experiments deploying GM-CSF-armed oncolytic adenoviruses in ATC show both tumor regression and concomitant immune stimulation, thereby validating progression to phase I clinical trials to interrogate safety and immune readouts *in vivo* (41, 50, 90).

While the aforementioned trials provide encouraging signals, it is imperative to interpret these results with caution. The majority of data regarding ATC and MTC are derived from Phase I/II single-arm cohorts with small sample sizes, making them susceptible to selection bias. Furthermore, the lack of randomized control arms in many studies complicates the differentiation between the true immunotherapeutic effect and the natural history of the disease in highly selected patient populations. Consequently, while current evidence supports the use of ICIs in advanced ATC (hypothesis-affirming), their role in DTC remains largely hypothesis-generating, necessitating validation through large-scale, randomized controlled trials

### 5.2 Response predictors and biomarkers

The strategic selection of patients most likely to benefit from immunotherapies depends on reliable predictive biomarkers (59), as mentioned.

(i)  PD-L1 Expression: Quantitative assessment of PD-L1 on tumor or infiltrating immune cells is positively linked to response to PD-1 checkpoint inhibition, particularly within the ATC subset (79, 115).
(ii)    Tumor Mutational Burden (TMB): A heightened TMB, observed more prevalently in ATC, correlates with a more extensive neoantigen reservoir and an attendant improvement in the clinical efficacy of immunotherapeutic agents (54, 111, 116).
(iii)    Immune Gene Signatures: Transcriptional profiles showing heightened expression of interferon-γ-related genes before treatment are enriched in patients experiencing favorable outcomes following exposure to checkpoint blockade (45, 117).
(iv)    RET and BRAF Mutations: These oncogenic lesions can modulate the immunogenic traits of the tumor and simultaneously serve as rational targets for antigen-directed immunotherapeutic interventions (82, 92, 97).

### 5.3 Real-world evidence

Despite the relative scarcity of randomized clinical trial data, observational practice has begun to illuminate the practical application of immunotherapy in rare, high-grade thyroid cancers (105, 118). Compassionate use of pembrolizumab in ATC has, when instituted early in the clinical course, been associated with rapid tumor shrinkage, notable extension of survival, and quantifiable gains in quality of life (6, 100). In select thyroid cancer patients, the strategic sequencing of ICIs with targeted kinase therapy has yielded disease stabilization that exceeds the durability associated with monotherapy of the latter (69, 97, 105). Collectively, these real-world data highlight the translational promise of immune-based strategies and encourage systematic exploration in carefully defined, biomarker-guided cohorts (59, 118).

## 6 Challenges and limitations

Although immunotherapy has made inroads in the treatment of thyroid cancers, its broader application remains constrained by a range of scientific, clinical, and logistical hurdles (108, 119).

### 6.1 Low immunogenicity in differentiated thyroid cancers

Differentiated thyroid carcinomas, notably papillary and follicular variants, display a low tumor mutational burden, a paucity of tumor-infiltrating lymphocytes, and a limited neoantigen landscape (64, 120). These attributes confer a cold immune phenotype that diminishes the effectiveness of ICIs (69, 98). Addressing this limitation necessitates the incorporation of approaches intended to elevate tumor immunogenicity, including localized radiation, selective kinase inhibitors, and oncolytic viral vectors, either sequentially or on a combinatorial basis (121–124). Addressing this limitation requires strategies to convert “cold” tumors into “hot” environments. For instance, the integration of radiotherapy can induce immunogenic cell death (ICD). This process releases damage-associated molecular patterns (DAMPs) and upregulates MHC-I expression on tumor cells, thereby facilitating the recruitment and infiltration of cytotoxic T lymphocytes. Concurrently, oncolytic viruses are being engineered to secrete cytokines like GM-CSF, further amplifying this systemic immune priming. For the vast majority of PTC and FTC patients, immunotherapy is currently defensible only within the context of clinical trials. The inherently low antigenicity of these tumors means that ICI monotherapy is unlikely to provide clinical benefit. Future success in this subgroup depends entirely on “immune-priming” strategies—such as combinations with TKIs or radiation—that can artificially inflame the microenvironment, rather than relying on pre-existing immunity.

### 6.2 Immune-related adverse events and endocrinopathies

Although durable remissions can accompany checkpoint blockade, the therapy is not devoid of risks (44). Immune-related adverse events may affect virtually any organ, yet endocrinopathies such as thyroiditis, subsequent hypothyroidism, adrenal insufficiency, and hypophysitis assume particular significance in the context of thyroid malignancy (100). These events can complicate the clinical course by destabilizing pre-existing hormonal dysregulation (102, 108). Effective mitigation hinges on prompt recognition, the integration of endocrinologists with oncologists, and the provision of comprehensive patient education that includes symptoms to be monitored (125). While manageable, these events are significant. Clinical observations indicate that immune-related thyroid dysfunction occurs in approximately 15–30% of patients receiving checkpoint inhibitors. Effective management requires a tiered protocol: asymptomatic or mild cases (Grade 1–2) typically allow for the continuation of immunotherapy with appropriate hormone replacement (e.g., levothyroxine for hypothyroidism), whereas severe inflammatory reactions (Grade 3–4) necessitate the temporary suspension of therapy and the administration of high-dose corticosteroids.

### 6.3 Resistance mechanisms

Immunotherapy for aggressive thyroid cancers faces both intrinsic and adaptive resistance (33, 67). Intrinsic or primary resistance often stems from barriers such as poor infiltration of activated T cells, defective MHC molecule expression (89), and a predominance of immunosuppressive elements, including regulatory T cells, myeloid-derived suppressor cells, and alternatively activated macrophages (126). Conversely, adaptive or acquired resistance evolves in response to pressure from therapy and may manifest as compensatory upregulation of inhibitory checkpoint receptors (127); most notably, T cell immunoglobulin and mucin-domain-containing molecule-3 and lymphocyte-activation gene 3, loss of the target antigen because of mutations or selective pressure, or a reorganized, immunosuppressive TME (31, 44). Elucidation of these escape pathways is essential to inform next-generation clinical strategies that incorporate rationally designed combination therapies (82, 128). Adaptive resistance often evolves under therapeutic pressure. A key mechanism involves the compensatory upregulation of alternative immune checkpoints, such as TIM-3 and LAG-3, following initial PD-1 blockade. This “checkpoint switching” limits T-cell effector function. Consequently, future clinical designs are increasingly focusing on dual-blockade strategies (e.g., anti-PD-1 plus anti-TIM-3) or the addition of MEK inhibitors to sensitize the tumor to immune recognition.

### 6.4 Limited clinical trial recruitment

The infrequency of aggressive thyroid neoplasms, specifically ATC and advanced MTC, complicates the speedy and sufficient recruitment of patients for investigational studies (105, 129). Geographic dispersion of a small cohort of patients, the absence of trial sites in resource-limited environments, and narrow eligibility definitions combine to constrain enrollment, which in turn delays the accrual of statistically powerful, high-quality clinical evidence (97, 108).

### 6.5 Translational gaps and biomarker limitations

Predictive biomarkers for immune checkpoint blockade in thyroid cancers have included tumor cell-surface PD-L1 expression, TMB, and select immune transcriptomic signatures (59, 79, 129). However, their performance for accurate patient stratification remains inadequate (130). The lack of standardized, validated biomarkers restricts the ability to individualize immunotherapy interventions (50). Furthermore, preclinical validation often utilizes immunocompromised murine models (67), which inadequately mirror the complexity of human tumor-immune system interactions, thereby limiting the translational value of observed therapeutic responses and contributing to clinical trial attrition (50, 88).

## 7 Future directions

### 7.1 Personalized immunotherapy through molecular and immune profiling

The extensive biological heterogeneity of thyroid cancers requires an immunotherapy platform based on precision medicine principles (31, 131). Multimodal profiling, spanning whole-exome sequencing (132), RNA sequencing (73, 133, 134), and mass spectrometry-based proteomics (127, 135), can map patient-specific neoepitopes, the full spectrum of somatic mutations (136), and pre-treatment immune microenvironments (137, 138). When assimilated into clinical workflows, these layers of information can guide the judicious selection of immunotherapeutic regimens, be it checkpoint inhibitors, engineered T cell therapies, or peptide-based vaccines, so that each intervention is calibrated to the distinct immunogenic and oncogenic profile of the tumor at hand (88, 94, 108, 139).

To translate these molecular insights into clinical practice, we propose a hierarchical biomarker framework:

(i)    Histology First: For ATC, immediate testing for BRAF V600E and PD-L1 is standard to guide the use of combinational therapies (e.g., Dabrafenib/Trametinib + Pembrolizumab).
(ii)    Tumor Mutational Burden (TMB) Assessment: For Refractory DTC, TMB testing is crucial. Patients with TMB-High (≥ 10 mut/Mb) are candidates for tissue-agnostic approval of pembrolizumab.
(iii)    Mismatch Repair (MMR) Status: Screening for dMMR/MSI-H identifies a small subset of responders eligible for immunotherapy.
(iv)    Investigational Stratification: Patients lacking the above markers (TMB-Low, MSS, BRAF-wt) should be directed toward clinical trials exploring immune-priming combinations (e.g., Lentatinib + ICI) rather than off-label monotherapy.

### 7.2 Neoantigen discovery and vaccine development

Recent improvements in next-generation sequencing and computational immunology have streamlined the characterization of neoantigens arising from non-synonymous mutations (40, 140). Machine learning (ML) algorithms can now evaluate peptide immunogenicity, enabling the selection of neoepitopes most likely to elicit durable T cell responses (141, 142). In the context of thyroid cancers, especially ATC, where a pronounced mutational burden is often present, such neoantigen-informed vaccines can function synergistically with ICIs by diversifying and sustaining the pool of tumor-specific cytotoxic T lymphocytes that can drive long-term disease control (6, 69, 95, 143, 144).

### 7.3 Modulation of the tumor microenvironment

Reprograming the TME to counteract immunosuppressive barriers remains a highly promising therapeutic strategy (31). Approaches currently under investigation include selective depletion or re-education of tumor-associated macrophages (67), neutralization of myeloid-derived suppressor cell activity (58, 66), antagonization of immunosuppressive cytokines such as TGF-β and IL-10, and augmentation of dendritic cell maturation (33, 145). Such interventions, when sequenced or co-administered with ICIs or ACT, have the potential to convert immunologically inert, well-differentiated thyroid cancers into immunologically active and responsive tumors (31, 63, 69, 146).

### 7.4 Microbiome-immune axis in thyroid cancer immunotherapy

Recent studies suggest the gut microbiome can influence both systemic immune homeostasis and the therapeutic response to immunotherapy (117, 147). Modulatory strategies, including dietary interventions, probiotics, and fecal microbiota transplantation, have been proposed to tilt systemic immune polarization toward a more pro-inflammatory profile, thereby augmenting therapeutic efficacy (148). Longitudinal studies of microbiome composition in thyroid cancer patients receiving immunotherapy are warranted to discern achievable microbial signatures that could serve as biomarkers or therapeutic targets (59, 149).

### 7.5 Next-generation cellular immunotherapies

Advances in cellular engineering are poised to overcome the limitations imposed by the TME (68). Next-generation CAR-NK cells (150), T-cell receptor–engineered T cells, and cytokine-secreting armored CAR-T cells can be tailored for enhanced specificity (56, 86, 151), sustained persistence, and durable resistance to immunosuppressive conditions (31, 59). The application of “safety switch” technologies in these constructs can constrain on-target, off-tumor toxicities, thereby safeguarding normal thyroid tissue and adjacent organs from collateral damage while retaining therapeutic efficacy against tumor cells (126, 131).

### 7.6 Rational multimodal integration

The promise of future iodine-resistant thyroid cancer management hinges on rational, multimodal constructs that integrate surgery (17, 152–154), small-molecule targeted inhibitors, radiotherapy, and immunotherapy into a unified therapeutic roadmap (49, 155, 156). For instance, a neoadjuvant immunotherapy phase could induce a downward stage migration in locally advanced disease, thereby enabling a more complete surgical resection while simultaneously entrenching durable immunological memory that guards against later relapse (101, 157). Subsequent radiotherapy and targeted kinase blockade might be temporally interleaved to unleash tumor-associated antigen exposure and amplifying immune activation, after which a prolonged immunotherapy maintenance phase would consolidate the antitumor effect (49, 98, 113, 131, 158).

### 7.7 Overcoming technological and logistical barriers in clinical translation

Despite the promising horizon of personalized immunotherapy, significant technological and logistical hurdles impede its widespread clinical adoption. Firstly, the “vein-to-vein” time interval remains a critical bottleneck. For patients with rapidly progressing malignancies like ATC, the weeks required to manufacture autologous CAR-T cells or personalized neoantigen vaccines may exceed their life expectancy. Consequently, a key transformation pathway lies in the development of “off-the-shelf” (allogeneic) cellular products and rapid-manufacturing vaccine platforms (e.g., mRNA technology) to ensure timely therapeutic accessibility. Secondly, bioinformatics standardization is urgently needed. While machine learning accelerates neoantigen discovery (as discussed in section 7.2), the lack of consensus on prediction algorithms leads to inter-institutional variability, hindering the validation of biomarkers across multi-center trials. Establishing harmonized computational pipelines will be a prerequisite for moving these precision tools from the laboratory to standard clinical practice.

## 8 Conclusion

Over the preceding decade, immunotherapy has evolved from a laboratory curiosity to an indispensable pillar of cancer care. In thyroid cancer, especially ATC and advanced MTC, the matrimonial incorporation of immune-engineered strategies rekindles optimism for overcoming previously unforgiving prognoses. The accelerating inventory of immune checkpoint antibodies, synthetic vaccines, engineered adoptive cell products, and oncolytic viral vectors attests to the intrinsic plasticity and breadth of immune pharmacology against endocrine neoplasms. Clinical outcomes, however, are not uniformly favorable among histological variants, with differentiated thyroid carcinomas exhibiting intrinsic limitations stemming from suboptimal immunogenicity and an immune-excluded TME. The evolving landscape of predictive biomarkers, specifically PD-L1 expression levels, TMB, and curated immune gene expression signatures, holds promise for enhancing biomarker-driven patient stratification (159), but definitive validation through expansive, long-term prospective cohorts remains a requisite. The documented onset of both primary and secondary resistance mechanisms further underscores the imperative for next-generation immunomodulatory agents and strategically conceived multimodal regimens tailored to the complex, heterogeneous mechanisms underpinning immune evasion.

Advancement in this domain will depend on the synergistic application of comprehensive molecular characterization, sophisticated bioinformatics pipelines, and adaptive clinical trial architectures designed to support precision-guided immunotherapy. As the pathological and immunological interplay within thyroid neoplasia is increasingly elucidated, the field is strategically positioned to expedite the clinical translation of preclinical insights into sustained therapeutic advantage (160). By simultaneously targeting pertinent oncogenic drivers and manipulating the immune microenvironment, contemporary immunotherapeutic strategies may progressively reengineer the clinical algorithm for advanced thyroid malignancies, migrating the treatment objective from ephemeral disease stabilization to durable remission and enhanced overall survival.
